# Hydrolyzed whey protein enriched with glutamine dipeptide attenuates skeletal muscle damage and improves physical exhaustion test performance in triathletes

**DOI:** 10.3389/fspor.2022.1011240

**Published:** 2023-01-06

**Authors:** Fabiano Pinheiro Peres, Adriana Cristina Levada-Pires, Marcelo Vieira, Elaine Hatanaka, Maria Fernanda Cury-Boaventura, Alessandra Folador, Renata Gorjão, Sandro Massao Hirabara, Paulo Roberto Santos-Silva, Patricia A. Deuster, Rui Curi, Tania Cristina Pithon-Curi

**Affiliations:** ^1^ Department of Physiology and Biophysics, Institute of Biomedical Sciences, Universidade de São Paulo, São Paulo, Brazil; ^2^ Institute of Physical Activity Sciences and Sports, Interdisciplinary Post-graduate Program in Health Sciences, Universidade Cruzeiro do Sul, São Paulo, Brazil; ^3^ Institute of Orthopedics and Traumatology, School of Medicine, Universidade de São Paulo, São Paulo, Brazil; ^4^ Department of Military and Emergency Medicine, Consortium for Health and Military Performance, F. Edward Hébert School of Medicine, Uniformed Services University of the Health Sciences, Bethesda, MD, United States

**Keywords:** skeletal muscle damage, physical exercise performance, second ventilatory threshold, maximal oxygen consumption, creatine kinase

## Abstract

**Purpose:**

To investigate the effects of hydrolyzed whey protein enriched with glutamine dipeptide on the percentage of oxygen consumption, second ventilatory threshold, duration and total distance covered, and skeletal muscle damage during an exhaustion test in elite triathletes.

**Methods:**

The study was a randomized, double-blinded, placebo-controlled, crossover trial. Nine male triathletes performed a progressive incremental test on a treadmill ergometer (1.4 km h^−1^·3 min^−1^) 30 min after ingesting either 50 g of maltodextrin plus four tablets of 700 mg hydrolyzed whey protein enriched with 175 mg of glutamine dipeptide diluted in 250 ml of water (MGln) or four tablets of 700 mg maltodextrin plus 50 g maltodextrin diluted in 250 ml of water (M). Each athlete was submitted to the two dietary treatments and two corresponding exhaustive physical tests with an interval of one week between the interventions. The effects of the two treatments were then compared within the same athlete. Maximal oxygen consumption, percentage of maximal oxygen consumption, second ventilatory threshold, and duration and total distance covered were measured during the exhaustion test. Blood was collected before and immediately after the test for the determination of plasma lactate dehydrogenase (LDH) and creatine kinase (CK) activities and lactate concentration (also measured 6, 10, and 15 min after the test). Plasma cytokines (IL-6, IL-1β, TNF-α, IL-8, IL-10, and IL-1ra) and C-reactive protein levels were also measured.

**Results:**

A single dose of MGln increased the percentage of maximal oxygen consumption, second ventilatory threshold duration, and total distance covered during the exhaustion test and augmented plasma lactate levels 6 and 15 min after the test. MGln also decreased plasma LDH and CK activities indicating muscle damage protection. Plasma cytokine and C-reactive protein levels did not change across the study periods.

**Conclusion:**

Conditions including overnight fasting and a single dose of MGln supplementation resulted in exercising at a higher percentage of maximal oxygen consumption, a higher second ventilatory threshold, blood lactate levels, and reductions in plasma markers of muscle damage during an exhaustion test in elite triathletes. These findings support oral glutamine supplementation's efficacy in triathletes, but further studies require.

## 1. Introduction

Blood glutamine levels range from 0.5 to 0.8 mM in healthy men and women. In skeletal muscle, glutamine represents about 60% of the total non-essential amino acid pool (1). However, glutamine deficiency can occur under extreme catabolic situations, such as cancer, overtraining, and severe illness, making it a conditionally essential amino acid (2, 3).

Stehle et al. (2017) reported through a meta-analysis study that critically ill patients supplemented with parenteral glutamine, following clinical guidelines (0.3–0.5 g/kg b.w./day; max. 30% of the prescribed nitrogen supply) as part of a balanced nutrition regimen, exhibit reduced hospital mortality, infectious complication rates, and hospitalization length (4). Moreover, Wiens et al. (2014) reported that 98% of athletes take at least one dietary supplement routinely, with glutamine being a notable one (5). Athletes consume protein powders, recovery drinks, branched-chain amino acids, and carbohydrates (CHO), like maltodextrin, to increase health, immune system function, and physical performance (6). It has also been shown that administering hydrolyzed protein enriched with glutamine increased soccer players’ performance during intermittent exercise (7). This dietary supplementation also protected leukocytes from apoptosis associated with one bout of exhaustive exercise in elite triathletes (8). Indeed, adding protein to a CHO supplement enhances physical performance beyond CHO alone (9–11). Khorshidi-Hosseini and Nakhostin-Roohi (2013) described that the combination of maltodextrin and glutamine is more effective at preventing anaerobic power reductions during repeated bouts of a Running-based Anaerobic Sprint Test protocol than CHO or glutamine supplementation separately (12).

Athletes and their physicians consistently report muscle injury, inflammation, and changes in leukocyte functions after the activities mentioned above (13, 14). Oral supplementation with dipeptide L-alanyl-L-glutamine (DIP) reduces muscle damage and inflammation in animal models (15–17). Cruzat et al. (2010) reported that rats submitted to swimming training for six weeks and then two hours of prolonged swimming exhibited decreased plasma CK and LDH activity when receiving DIP supplementation (1.5 g·kg^−1^, for 21 days before euthanasia) (15). Rats submitted to progressive resistance exercise for eight weeks and supplemented with DIP for 21 days before euthanasia also showed lower plasma levels of CK and LDH activities compared to non-supplemented rats. Others also described the beneficial effects of glutamine, after 21 days of supplementation, in an exercise-induced injury animal model (16, 17). In athletes, studies showing a protective effect of glutamine on muscle damage are scarce. Córdova-Martínez (2021) reported a reduction in CK and LDH activities after 20 days of glutamine supplementation in basketball players (18). In the studies mentioned above, the effect of glutamine was observed after its administration for 20–21 days; however, the protective impact of a single dose of this amino acid on triathletes’ muscle damage remains to be investigated.

There are diverse types of triathlon competition energy expenditure tests, and the average test time depends on competition characteristics. For example, athletes frequently participate in short (0.75 km swimming, 20 km cycling, and 5 km running), Olympic (1.5 km swimming, 40 km cycling, and 10 km running), Half Ironman (2 km swimming, 90 km cycling, and 21 km running), or Ironman (3.8 km swimming, 180 km cycling, and 42.2 km running) triathlon races. The duration of the event is vital to metabolic and nutritional concerns for working muscles and energy expenditure, but few studies investigated this competition in field conditions. Barrero et al. (2014) studied energy expenditure and fluid balance in eleven triathletes through the ultra-endurance triathlon (mean competition time was 755 ± 69 min). They estimated energy expenditure at 11,009 ± 664 kcal and mean energy intake at 3,643 ± 1,219 kcal. These authors concluded that the high energy demands of this race type result in a large energy deficit after the race if not compensated by nutrient and fluid intake (19). In this sense, strategies to minimize this deficiency can improve athlete performance. In a case study, Gillum et al. (2006) reported that one male triathlete (38 years old) completed the Half Ironman competition and had an estimated CHO energy expenditure of 10,003 kJ for the bike segment and 5,759 kJ for the run segment of the race (20). During the race, the athlete consumed 308 g of exogenous CHO (liquid and gel; 1.21 g CHO/min), and muscle glycogen decreased from 227.1 pre-race to 38.6 mmol. kg wet weight^−1^ one-hour post-race. Additionally, Kimber et al. (2002) reported a mean energy intake of 16,500 kJ and an estimated energy expenditure of 42,050 kJ for male triathletes during a full Ironman triathlon (21).

Coqueiro et al. (2018) demonstrated that glutamine dipeptide supplementation increases ammonia and glycogen concentrations in skeletal muscle, and glutamine plus alanine in their free form prevents the muscle ammonia increase during resistive exercise training (22). A meta-analysis study described that glutamine supplementation has no marked effect on athletics’ immune system, aerobic performance, or body composition. However, the authors suggested that the efficacy of glutamine supplementation on blood neutrophil number depends on the supplement type and dose (23).

Herein, we investigated the effects of hydrolyzed whey protein enriched with glutamine dipeptide and maltodextrin on exhaustive physical test performance in triathletes. We compared the effects of a single dose of a combination of hydrolyzed protein supplemented with glutamine and maltodextrin or maltodextrin only, administered once, on elite triathletes’ performance and muscle injury during an exhaustive physical test. We hypothesized that a single dose of whey protein-enriched glutamine dipeptide supplementation could improve functional performance and reduce muscle damage in triathletes. We investigated the effects of hydrolyzed protein enriched with glutamine on the percentage of oxygen consumption, second ventilatory threshold, duration, and total distance covered during a physical exhaustion test in triathletes. We also assessed indicators of skeletal muscle (lactate dehydrogenase and skeletal muscle creatine kinase activities) and heart tissue (myocardial creatine kinase activity) damage and plasma cytokine levels.

## 2. Methods

### 2.1. Subjects

Nine healthy elite Caucasian male triathlon athletes signed an informed consent form agreeing with the procedures described for this study. The Ethical Committee of the Institute of Biomedical Sciences, University of São Paulo, approved the experimental protocol (number 694/CEP). The athletes in our study were top of their age in the Brazilian ranking in triathlon competitions and won races from their age category. All procedures were performed following the Declaration of Helsinki. The characteristics (mean ± SD) of the athletes were as follows: age: 24.9 ± 4.0 years old; body mass: 69.3 ± 4.7 kg; height: 1.78 ± 0.06 m; body fat percentage: 8.6% ± 1.2%; and VO_2_max 63.52 ± 3.85 ml·kg^−1^·min^−1^. The volume of training hours of the triathletes in the present study was three hours per day, six times per week, making a total of 18 h per week, which can be considered high performance. According to interview exclusion criteria, athletes must have competed in this modality for at least five years. The triathletes were non-smokers, and none of them was taking any medication at the time of testing. We previously used the same athletes under the same conditions to examine the exhaustive physical test's effect on neutrophil and lymphocyte death (8).

### 2.2. Experimental design, dietary supplement interventions, and anthropometric measurements

Athletes did not use supplements during the training and the weeks before the study. Subjects were instructed to maintain similar training and diet regimens during the days before participation in the study. One can find details of the design and supplementation in our previous study (8). The authors documented all information concerning individual exercise training programs, maintained dietary habits, dietary supplementation, and endurance test. The study was a randomized, double-blinded, placebo-controlled, crossover trial. After an overnight fast, to avoid large discrepancies in the feeding state, the athletes took four tablets of 700 mg of hydrolyzed whey protein enriched with 175 mg glutamine dipeptide (total glutamine = 700 mg, manufactured by DMV International Veghel, Netherlands) plus 50 g maltodextrin (Carb Up^@^, manufactured by Probiótica, Brazil), diluted in 250 ml water (MGln). This dose led to a significant increase in glutamine plasma levels after 30 min. The same dosage and supplementation protocol were previously used (8). Testing started around 9 a.m. The glutamine dosage used is within the range of the dietary supplementation the athletes usually use. The M supplementation consisted of four tablets of 700 mg maltodextrin and 50 g of maltodextrin diluted in 250 ml water (total maltodextrin = 52.8 g). The supplements, identical in appearance and taste, were prepared immediately before administration and given 30 min before the exhaustive exercise test started. We submitted each athlete to the two treatments (MGln and M) and two corresponding physical exhaustion tests with an interval of one week between the interventions.

The authors followed the recommendations of the International Society for the Advancement of Kinanthropometry (ISA) to perform the measurements of total body mass (Kg), stature (m), subcutaneous fat, and body circumference (24, 25).

### 2.3. Endurance test

Aerobic power and aerobic capacity tests were performed on a motorized treadmill. The athletes ran on a 1% grade, and oxygen consumption (VO_2_) was continuously measured using a Vmax 29 (SensorMedics, United States). Before each test, the flow sensor (using a syringe of a 3 L volume) and O_2_ and CO_2_ sensors (using a known gas mixture, 16% O_2_, 4.01% CO_2_, and balanced with nitrogen) were calibrated (26). The triathletes rested for 2 min, and their heart rate was determined. Subsequently, the athletes exercised for 3 min at 6, 7, and 8 km/h, one minute at each velocity. The test was initiated at 9.4 km/h (called stage one), and the speed was increased in each stage by 1.4 km/h every 3 min until they reached volitional fatigue. After the test, the athletes recovered for 4 min at 6, 5, and 4 km/h before resting. Blood pressure was measured manually before the test, every 2 min during the test, immediately after exercise cessation, during the first minute of recovery and every 2 min after that. The test was undertaken in normothermic laboratory conditions (20–22 °C, 40%–60% RH). The subjective perception of their effort was determined for each test minute using Borg's 15-point linear scale rating of perceived exertion (27). Verbal encouragement for them to perform up to their maximum limits was provided throughout the test. The test was terminated when the athletes could not keep pace with further increments in exercise intensity. A similar description can be found in our previous study (8).

### 2.4. Cardiopulmonary exercise testing

Ventilatory equivalents (VE), oxygen consumption (VO_2_), carbon dioxide production (VCO_2_), and respiratory exchange ratio (RER) were continuously monitored through a breath-by-breath system as described by Favano et al. (2008) (7). The signs of exhaustion adopted included: extremely forced ventilation, fatigue, facial flushing, dyspnea, and unsteady gait. The second ventilatory threshold (VT_2_) was determined using the following criteria: (a) loss of linearity between VE and VCO_2_, according to the lowest value of the VE of carbon dioxide (VE/VCO_2_); (b) the highest value of the expired fraction of carbon dioxide (FECO_2_), with subsequent abrupt rises (c) in VE, (d) respiratory rate and (e) tidal volume plateau (28). During the test, the athletes had a rubber mouthpiece and nose clip and overcame the loss of verbal communication with a subject during the test with prearranged hand signals. Heart rate with a continuous 12-lead electrocardiogram (ECG) was monitored and measured each minute utilizing a computerized ECG (HeartWare, 6.4, BH, Brazil). A maximal effort level was considered when met two of the following five criteria: (i) plateau when the difference in the VO_2_ in the last two stages of the incremental test was ≤2.1 ml·kg^−1^·min^−1^ (29); (ii) maximal HR within 5 b/min (95%) of the age-predicted maximum [208 – (0.7* age)] (30); (iii) volitional fatigue; (iv) more than 18 on subjective Borg's scale (31); and (v) subjective indications such as sweating, hyperpnea, and facial flushing (32). Data from the VO_2_max tests were time-averaged using 30-s intervals.

### 2.5. Blood sample collection

Blood samples were collected from eight healthy male triathlon athletes at the top of their age category. One participant did not feel well during blood collection. Blood was collected from the antecubital vein into vacuum tubes containing an anticoagulant (0.004% ethylenediaminetetraacetic acid (EDTA) before supplementation and immediately after the exhaustion test. Afterward, whole blood was centrifuged at 500 × g for 10 min at 4 °C to obtain the plasma. Samples were separated and frozen (−70 °C) to assess cytokines, C-reactive protein, creatine kinase (total CK and myocardial CK isoenzyme - CK-MB), and lactate dehydrogenase activities. Plasma CK and LDH activities are indirect markers of muscle damage.

### 2.6. Blood measurements

Blood samples were centrifuged at 800 × g for 10 min, and the plasma was separated and maintained at −80 °C until measuring the cytokine concentrations. Lactate was determined in the blood collected from the fingers with a YSI 1,500 analyzer (Yellow Springs Instruments, Yellow Springs, OH, United States).

CK and LDH activities were measured according to the methods established by Oliver and colleagues (1955) (33) and Zammit and Newsholme (1976) (34), respectively.

Skeletal muscle CK (CK-MM) activity values were calculated by subtracting the plasma CK-MB values from the total CK activities. Plasma CK and LDH activities were expressed as U/L.

Plasma concentrations of IL-6, IL-1β, TNF-α, IL-8, IL-10, and IL-1ra were determined using Duoset enzyme-linked immunosorbent assay (ELISA), Duoset Kit of R&D System (Quantikine, R&D System, Minneapolis, MN, United States) according to the manufacturer's instructions. The intra-assay coefficient of variation (CV) was 4.1%–10%, the inter-assay CV was 6.6%–8.0%, and the sensitivity (pg/mL) was: 4.68 for IL-6, 1.95 for IL-1β, 7.81 for TNF-α, 15.62 for IL-8 and IL-10, and 19.53 for IL-1ra. C-reactive protein was measured using a latex-enhanced immunoturbidimetric assay capable of measuring protein levels within and outside the normal range (Bioclin Diagnostics, Belo Horizonte, MG, Brazil). The C-reactive protein concentration was determined by measuring the optical density variation at 505 nm in duplicate. The intra-assay CV was 1.6%–4.0%, the inter-assay CV was 0.6%–1.5%, and the sensitivity was 0.0312 mg/mL for C-reactive protein.

### 2.7. Statistical analysis

#### 2.7.1. Sample size estimations

To test the hypothesis of a difference in CK between groups, we considered an activity greater than 50 U/L significant. Galan 2017 (35) reported a standard deviation of around 70 U/L in CK activity. The present study was performed in high-level competitive triathletes; therefore, a lower standard deviation (35 U/L of CK activity) was considered for this specific group. For this reason, alpha was set at 0.05, and power was set at 0.8. Thus, at least seven triathletes were required for this study.

### 2.8. Data analysis approaches

Quantitative variables are presented as the mean and standard deviation or median and quartile intervals. Qualitative variables are presented as absolute and relative frequencies. We compared interventions (M and MGln) using the paired Student *t*-test. However, when the normality assumption was not met, the paired Wilcoxon test was used. The means on Borg's scale and heart rate for the M and MGln groups were compared in each stage of the endurance test. The lactate dehydrogenase, total creatine kinase, muscle creatine kinase, and cytokine production were analyzed by ANOVA for repeated measures when the assumption of normality was met; otherwise, the KS (Kolmogorov–Smirnov) and Friedman tests were used when multiple comparisons detected significance between moments. The level of significance was 0.05. The data analysis was performed using SPSS, version 19 (Statistical Package for the Social Sciences) for Windows and R core 3.4.

## 3. Results

**Figure 1:**
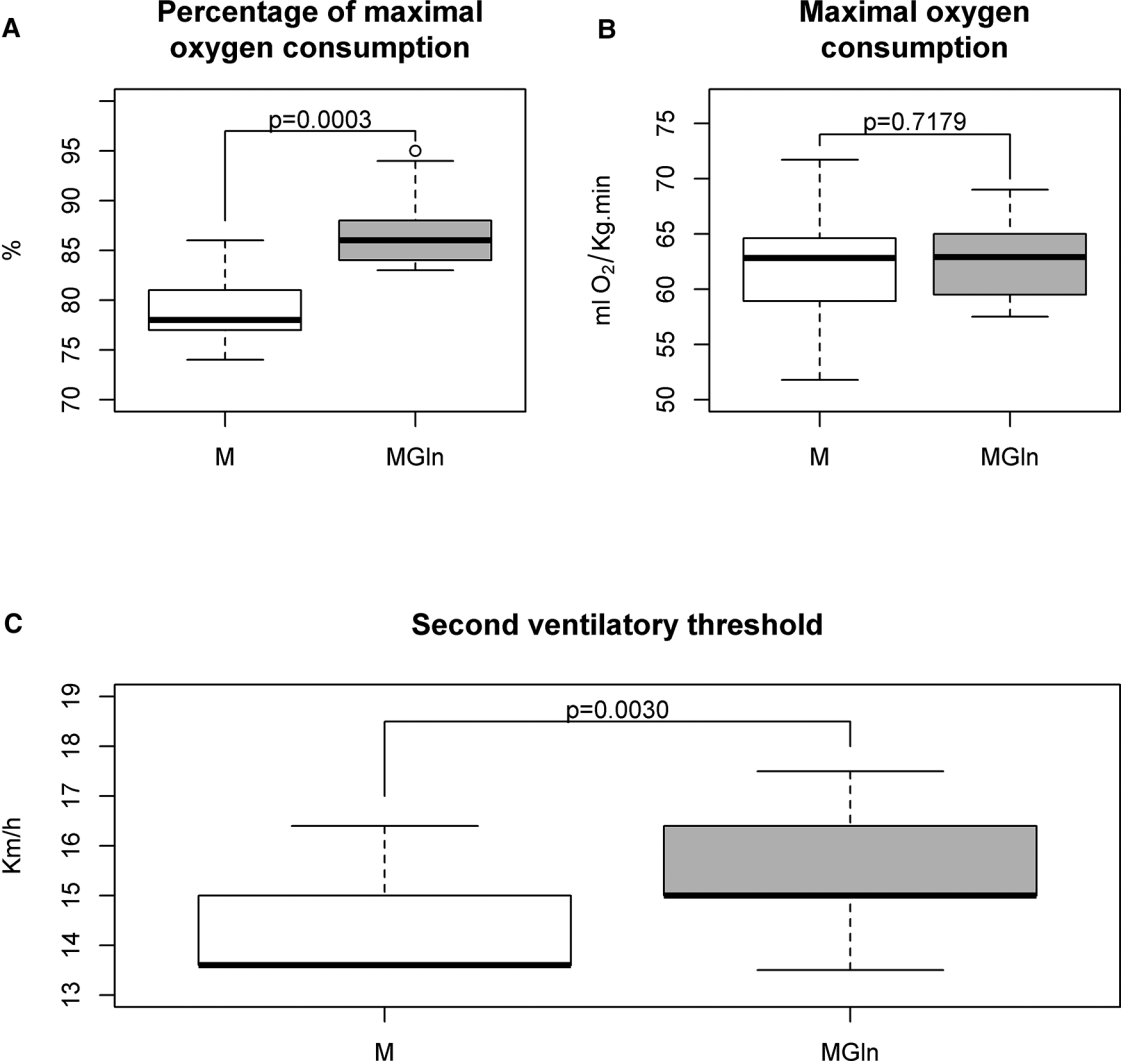
Cardiopulmonary testing in athletes supplemented with maltodextrin (M) or maltodextrin plus hydrolyzed protein enriched with glutamine (MGln). Percentage of maximal oxygen consumption (**A**), maximal oxygen consumption (**B**), and second ventilatory threshold (**C**) from athletes supplemented with M or MGln. For both tests, a significance level of 0.05 was set.

As reported in our previous study, the experimental protocol used for dietary supplementation ensured a significant increase in plasma glutamine concentration but did not change plasma glucose (8). Maximum oxygen consumption did not differ between M and MGln treatments (M 62.0 ± 6.3 ml·kg^−1^·min^−1^ vs. MGln 62.7 ± 3.7 ml·kg^−1^·min^−1^) (Figure 1B). Nonetheless, as indicated by the maximum oxygen consumption percentage, the relative exercise intensity was 10% higher with the MGln than the M supplementation (M 79.3% ± 4.3% vs. MGln 87.2% ± 4.5%) (Figure 1A). The second ventilatory threshold (VT_2_) was 8.3% higher in the MGln than the M treatment (M 14.4 ± 1.0 Km/h vs. MGln 15.6 ± 1.19 Km/h) (Figure 1C).

**Figure 2:**
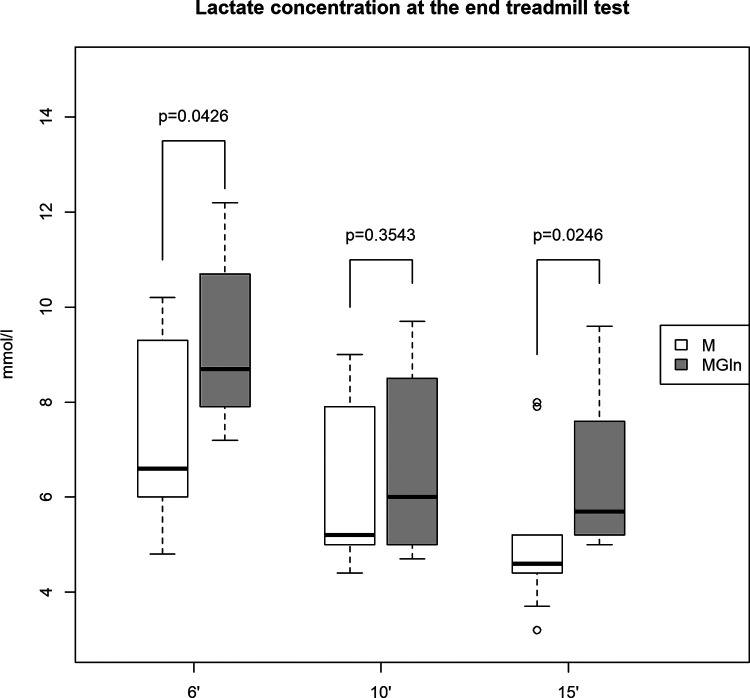
Plasma lactate concentrations in athletes supplemented with maltodextrin (M) or maltodextrin plus hydrolyzed protein enriched with glutamine (MGln). Plasma lactate concentration (mmol/L) at 6, 10, and 15 min after exercise in athletes supplemented with M or MGln. For both tests, a significance level of 0.05 was set.

Blood lactate levels were 25% higher in the MGln than the M supplementation 6 (M 7.5 ± 2.1 mmol/L vs. MGln 9.4 ± 1.8 mmol/L) and 15 (M 5.2 ± 1.7 mmol/L vs. MGln 6.5 ± 1.7 mmol/L) minutes after the test (Figure 2). However, no significant difference in plasma lactate concentrations was detected 10 min after the test (Figure 2).

**Figure 3:**
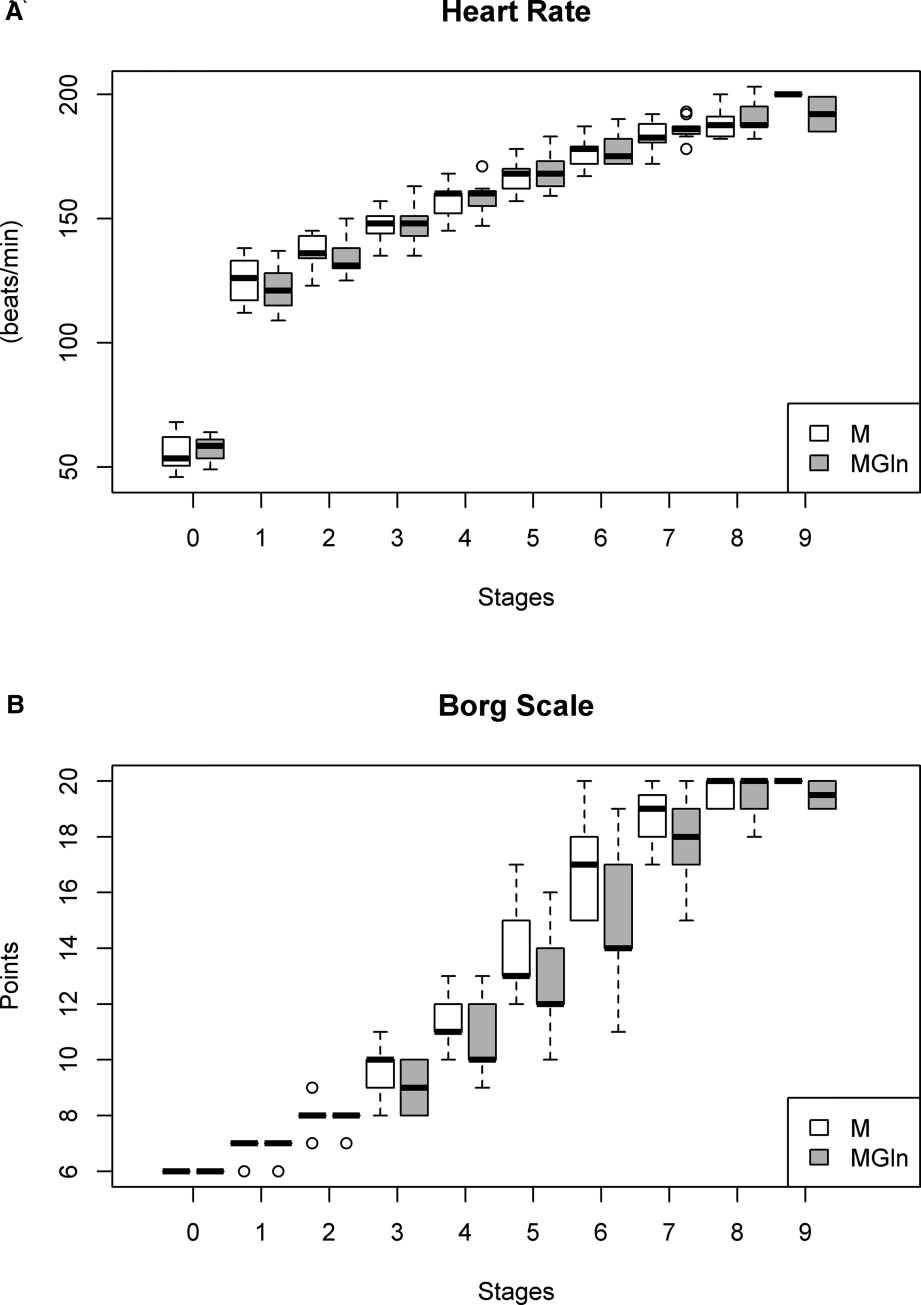
Heart rate (**A**) and borg scale (**B**) in athletes supplemented with maltodextrin (M) or maltodextrin plus hydrolyzed protein enriched with glutamine (MGln). Aerobic power and aerobic capacity tests were performed on a motorized treadmill. The athletes rested for 2 min, and their heart rate was determined. Then the athletes exercised for 3 min at 6, 7, and 8 km/h, one minute for each velocity. The test was initiated at 9.4 km/h (stage one), and the speed was increased by 1.4 km/h every 3 min until volitional fatigue was reached. The athletes ran on a 1% grade, and oxygen consumption (VO_2_) was continuously measured. The subjective perception of the effort was determined for each stage using Borg's 15-point linear scale rating of perceived exertion. For both tests, a significance level of 0.05% was set.

Heart rate was elevated proportionally relative to intensity during the exercise, and the supplement interventions did not alter this relationship (Figure 3A). The heart rate response at peak exertion for both tests in the two conditions, M and MGln, were maximal for absolute (M 192 ± 8 vs. MGln 190 ± 6 bpm; K-S = 0.148 and 0.163) and relative values (M 100 ± 4 vs. MGln 98% ± 3%; K-S = 0.238 and 0.259). In both conditions, the triathletes exceeded 95% of the HRmax predicted for age. Parasympathetic activation indicated by a reduction in the difference between maximum heart rate and heart rate attained in the first minute of recovery between the two conditions (M 38 ± 11 vs. MGln 39 ± 10 bpm) was not different (*p* = 0.793). The subjective perception of the effort (Borg scale) also did not differ between the two-supplementation treatments (Figure 3B).

**Figure 4:**
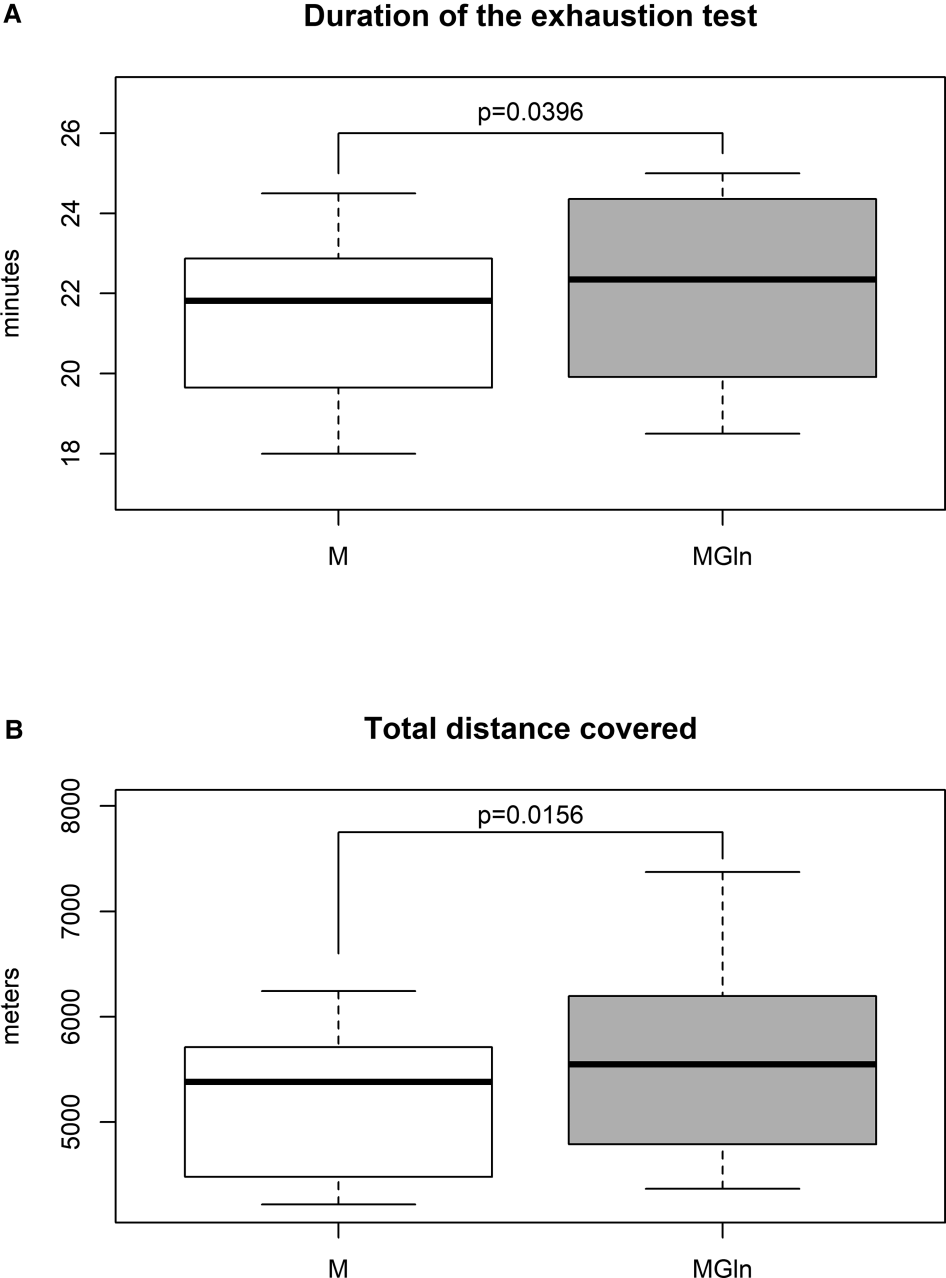
Duration time (**A**) and total distance covered (**B**) of the exhaustion test in athletes supplemented with maltodextrin or maltodextrin and hydrolyzed whey protein enriched with glutamine (MGln). Aerobic power and aerobic capacity tests were performed on a motorized treadmill. The athletes rested for 2 min, and their heart rate was determined. Then the athletes exercised for 3 min at 6, 7, and 8 km/h, one minute for each velocity. The test was initiated at 9.4 km/h (stage one), and the speed was increased by 1.4 km/h every 3 min until volitional fatigue was reached. The athletes ran on a 1% grade, and oxygen consumption (VO_2_) was continuously measured. For both tests, a significance level of 0.05 was set.

**Figure 5:**
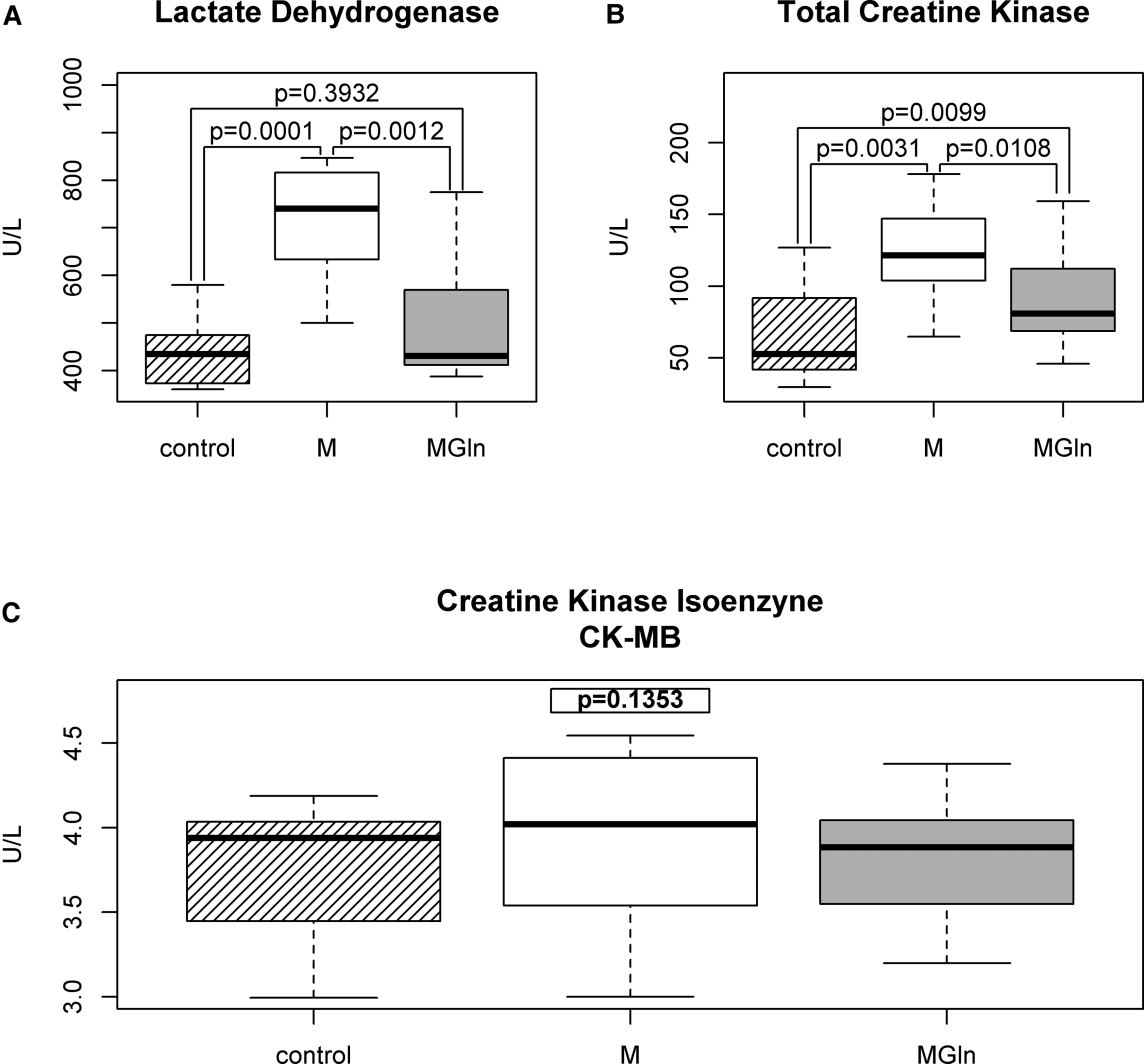
Muscle damage in athletes supplemented with maltodextrin (M) or maltodextrin plus hydrolyzed protein enriched with glutamine (MGln). Plasma activities of lactate dehydrogenase (**A**), total creatine kinase (**B**), and myocardial creatine kinase isoenzyme (MB-CK) (U/L) (**C**) in the periods before the tests and after maltodextrin (M) or maltodextrin and hydrolyzed whey protein enriched with glutamine (MGln) supplementation immediately after the end of the endurance test. For both tests, a significance level of 0.05 was set.

The duration of the exhaustion test increased by 3.3% (M 21.4 ± 2.2 vs. MGln 22.1 ± 2.5 min) (Figure 4A), and the total distance covered increased by 2.8% (M 5,201.9 ± 744.6 vs. MGln 5,601.5 ± 995.7 m) (Figure 4B) were significantly higher in the MGln compared to the M treatment. The MGln supplementation reduced physical effort-induced muscle damage, as indicated by reduced plasma activities of LDH (at rest 437.9 ± 74.2 U/L, M 715.8 ± 125.8 U/L and MGln 497.9 ± 146.4 U/L, a 30.4% decrease) (Figure 5A), total CK (at rest 66.1 ± 34.2 U/L, M 123.4 ± 35.1 U/L and MGln 91.0 ± 36.2 U/L, reduced by 26.2%) (Figure 5B), and skeletal muscle CK (at rest- 62.4 ± 34.1 U/L, M- 119.5 ± 35.1 U/L and MGln 87.3 ± 35.9 U/L, a 26.9% decrease) compared with the M treatment. The CK-MB activity did not differ between the two treatments (at rest 3.8 ± 0.4 U/L, M 3.9 ± 0.6 U/L and MGln 3.82 ± 0.4 U/L) (Figure 5C). Furthermore, no changes in pro- or anti-inflammatory cytokines or C-reactive protein plasma concentrations were observed (data not shown).

## 4. Discussion

Glutamine is an essential amino acid during periods of catabolic stress (36, 37), and its administration has been reported to reduce muscle injury in rats (15–17). Compared to M only, MGln supplementation resulted in the physical effort at a higher percentage of maximal oxygen consumption, improved second ventilatory threshold, increased duration and distance covered during the exhaustion test, and reduced circulating markers of muscle injury. Sinisgalli et al. (2021) reported that athletes training more than 20 h per week (high volumes) forty days before the race do not exhibit increased performance (overall race time) compared to those who train up to 14 h per week (lower volumes) (38). Parasympathetic reactivation, characterized by a reduction in heart rate in the first minute after intense exertion, has been used to address athletic performance. Indeed, rapid cardiorespiratory and metabolic recovery is closely associated with better athletic performance. In the present study, when comparing the two supplementations (M and MGln), there was no significant difference in the HR decrease. A drop in HR of 38 and 39 beats, respectively, in the first minute of recovery (HHR1), was verified in all triathletes, which is consistent with well-trained endurance athletes (39). HRR1 min could be indirectly used as an index of aerobic capacity, irrespective of age (39). A predominance of sympathetic tone may be associated with an increased risk of arrhythmias (40). Therefore, triathletes exhibited a low cardiovascular risk. These results indicate that MGln has favorable performance-related properties and confirm the study hypothesis.

Herein, MGln supplementation increased blood lactate levels at 6 and 15 min following the completion of the exhaustion test, possibly due to glutamine-stimulated anaerobic glycolysis (41). The utilization of glutamine by the muscle generates glutamate and α-ketoglutarate, which enter the Krebs cycle to produce oxaloacetate. This latter metabolite combines with acetyl-CoA to produce citrate through citrate synthase activity. Pyruvate is formed through glycolysis from glucose or glycogen and is the precursor for oxaloacetate and acetyl-CoA. The provision of oxaloacetate by glutamine then reduces this metabolite requirement from pyruvate. Consequently, pyruvate is spared and redirected to lactate formation. Alternatively, malate generated through glutaminolysis can be transported from the mitochondria to the cytosol and converted to pyruvate *via* the malic enzyme (42–44).

On the other hand, glutamine contributes to hepatic gluconeogenesis by competing with the lactate remaining in the circulation. The final result would be increased blood lactate concentrations (45). This latter effect would become less pronounced over time due to lowering blood glutamine levels. Some muscle fiber types also interfere with lactate clearance during rest (46). For example, athletes with a higher percentage of type I muscle fibers (aerobic) have an increased capacity for lactate clearance through oxidation than athletes whose muscle fibers are mostly type II (anaerobic) (47). The attenuating effect of the pretreatment with L-glutamine on skeletal muscle atrophy induced by 24-h fasting varies with the skeletal muscle fiber type. Soleus muscle, composed mostly of type I fibers with an intense oxidative metabolism, showed a more pronounced intracellular L-glutamine turnover than EDL muscle (composed mainly of type II fibers) after L-glutamine supplementation (36). Our study agrees with previous findings where adding protein to a CHO dietary supplement enhanced endurance performance compared to a 6%–10% CHO supplement (9–11). Favano et al. (2008) also administered glutamine and maltodextrin combined and reported a positive effect in increasing the distance covered and the time of tolerance in the feeling of fatigue to intermittent exercise in soccer players (7). Levenhagen and colleagues (2002) reported that protein added to a CHO supplement (3% CHO plus 0.75% protein or CHO 4.5% plus 1.2% protein) maintains endurance performance relative to the usual 6%–10% CHO supplement (48). McCleave et al. (2011) described that a 4.5% CHO plus 1.2% protein supplementation and a 3% CHO plus 0.75% protein mixture improve endurance performance compared to a 6% CHO sports drink in trained female athletes (9). Khorshidi-Hosseini and Nakhostin-Roohi (2013) reported that combining glutamine and maltodextrin two hours before exercise improves athletes’ physical performance during repeated competitions (12). Additionally, in rats, our group demonstrated that glutamine supplementation mimics some effects of overload on extensor digitorum longus (EDL) hypertrophy and that the association of glutamine and overload-induced EDL muscle hypertrophy led to a further increase in the resistance to fatigue (49).

The skeletal muscle glutamine pool is significantly reduced under certain metabolic stress conditions such as injury and burn (50) and during/after strenuous exercise (51, 52). The supplementation with alanyl-glutamine dipeptide increases plasma glutamine concentrations 45 min after ingestion and remains elevated for up to 60 min afterward (53, 54). In our previous study, plasma glutamine levels were increased after MGln athletes finished the exhaustion test (7). Therefore, MGln supplementation may minimize the consequences of reducing plasma glutamine levels.

Fatigue during sprint and endurance exercise is associated with glycogen depletion (53). Supplementation with CHOs and whey protein activates key skeletal muscle enzymes that regulate glucose metabolism and glycogen synthesis, which could attenuate exercise-induced glycogen depletion (55). It was also demonstrated that Gln was associated with efficient mitochondrial activity in muscle cells (56). It is plausible that MGln supplementation reduces skeletal muscle glycogen depletion by generating skeletal carbon molecules, which would then be available for anaerobic and aerobic metabolic pathways. Glutamine dipeptide supplementation increased glycogen concentrations in muscle and ammonia in the liver and muscle. Also, elevated free tryptophan/total tryptophan ratio, hypothalamic serotonin, and the serotonin/dopamine ratio in rats supplemented for 21 days and submitted to resistance training (22).

Athletes often complain about skeletal muscle pain during and after a triathlon competition. Pain during physical exercise occurs due to muscle injury (57) and can decrease performance. Runners supplemented for seven days with glutamine (1.5 g/kg glutamine + 250 ml water + 15 g sweetener) after running 14 km exhibited a decrease in muscle damage and oxidative stress indicators (58). The study by Córdova-Martinez et al. (2021) on basketball players indicates that glutamine administration (6 g per day for 20 days) attenuates exercise-induced muscle damage in a sport modality with a predominance of eccentric exertions (18). Street et al. (2011) investigated ingestion of 0.3 g kg^−1^ body mass of maltodextrin dissolved in 750 ml lemon flavored water (0.3 g kg^−1^ L-glutamine) at 0, 24, 48, and 72 h post an eccentric exercise and found that glutamine supplementation attenuated muscle strength loss, soreness and muscle damage (59).

As expected, plasma activities of total creatine kinase (CK-MM) and LDH were increased immediately after the exhaustion test (60). MGln supplementation attenuated the increase in total creatine kinase, CK-MM, and LDH activities in plasma from triathletes even after a single dose of MGln, indicating that this supplement attenuated muscle damage during the endurance test. This result is consistent with Saunders et al., who reported that CHO plus protein supplementation decreases muscle injury post-cycling exercise compared to CHO only and noted that CHO plus protein supplementation enhances the time to exhaustion (10, 11).

Supplementation with alanyl-glutamine dipeptide also attenuated muscle damage in rats subjected to prolonged exercise (15). The protective effect of glutamine supplementation on muscle damage may be partially explained by the fact that exhaustive aerobic exercise causes oxidative stress (61). The antioxidant systems, such as glutathione (GSH), are important for limiting muscle damage, and antioxidant capacity may be enhanced by glutamine supplementation (62, 63). Glutamine is a precursor for glutathione synthesis (41) and serves as a precursor in various biosynthetic pathways rather than serving as fuel (42). Corroborating with these findings, our group previously demonstrated that oral supplementation of L-glutamine for 30 days improves the strength and power of knee muscles in association with improved glycemia control and concomitant boost of plasma antioxidant capacity of older exercising women (64).

We did not detect cytokine plasma level changes immediately after the endurance test, as one would predict for the period studied (65). However, we did observe a glutamine cytoprotective effect. The cytoprotective effect of chronic glutamine supplementation on damaged tissue is associated with an increase in HSP 70 levels in skeletal muscle and PBMC (human peripheral blood mononuclear cells). HSP 70 reduces the DNA-binding activity of NF-κB, inhibiting inflammatory cytokine production and muscle damage induced by heavy resistive exercise (17). There is evidence that HSP 70 has an essential role as a regulator of the early inflammatory response to muscle injury, participating in myofiber regeneration and recovery (66).

The readers must consider some particularities of this study. We evaluated nine healthy elite male triathlon athletes at the top of their age category in the Brazilian rankings, making this a very homogenous sample. We compared the effects of the same athlete's two treatments to reduce large result variations. This protocol facilitated the detection of significant MGln effects and may account for why others, using a different protocol, did not observe these glutamine supplementation impacts (23, 67). The study conditions included overnight fasting to avoid significant discrepancies in the feeding state. The conditions did not change the plasma glucose concentration at the pre-test, which was within the normal range, i.e., 100–110 mg/dl. Supplementation with MGln did not change plasma glucose levels as compared to M only, as reported in our previous study (8). This finding indicates that hepatic glycogenolysis was not different among participants. The athletes had a similar glycemic homeostasis condition at the beginning of the test.

**Figure 6:**
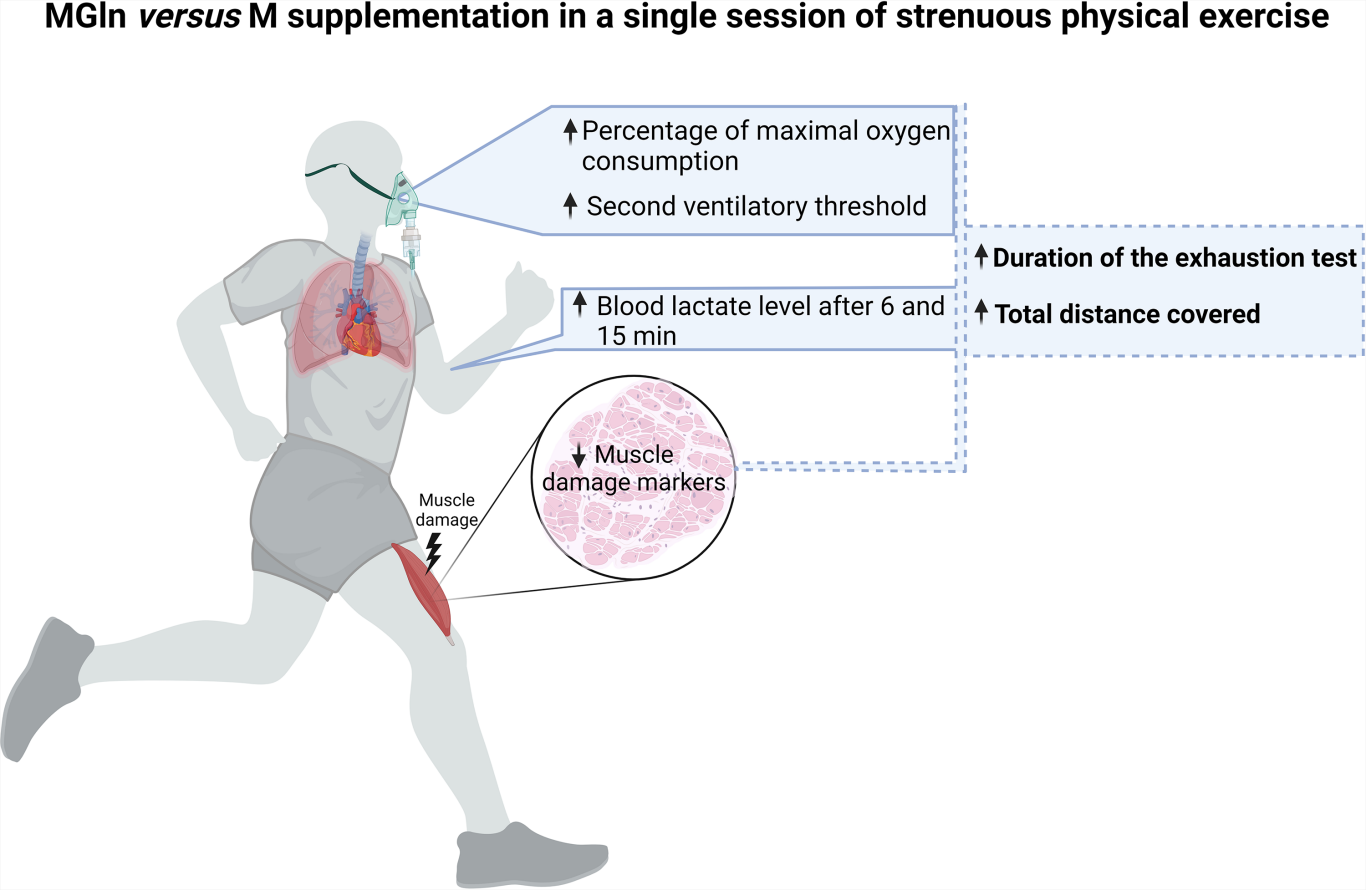
Graphical abstract – A single dose of MGln supplementation resulted in exercising at a higher percentage of maximal oxygen consumption, a higher second ventilatory threshold, blood lactate levels, and reductions in plasma markers of muscle damage (LDH, total CK and skeletal muscle CK). Created with BioRender.com.

We concluded that conditions including overnight fasting and a single dose of MGln supplementation resulted in exercising at a higher percentage of maximal oxygen consumption, a higher second ventilatory threshold, blood lactate levels, and reductions in plasma markers of muscle damage (Figure 6). Glutamine administration preserved the triathletes’ physical capacity, partially mitigated muscle damage, and contributed to a small but significant increase in the physical exhaustion test duration. These findings support oral glutamine supplementation's efficacy in triathletes, but further studies require.

## Data Availability

The raw data supporting the conclusions of this article will be made available by the authors, without undue reservation.
